# The role of deleterious mutations in the stability of hybridogenetic water frog complexes

**DOI:** 10.1186/1471-2148-14-107

**Published:** 2014-05-16

**Authors:** Pasquale Bove, Paolo Milazzo, Roberto Barbuti

**Affiliations:** 1 Dipartimento di Informatica, Università di Pisa, Largo B. Pontecorvo, 3, 56127 Pisa, Italy; 2 Museo di Storia Naturale, Università di Pisa, Via Roma, 79, 56011 Calci (Pisa), Italy

**Keywords:** Hybridogenesis, Water frogs, Sexual selection, Computational models, Simulations

## Abstract

**Background:**

Some species of water frogs originated from hybridization between different species. Such hybrid populations have a particular reproduction system called hybridogenesis. In this paper we consider the two species *Pelophylax ridibundus* and *Pelophylax lessonae*, and their hybrids *Pelophylax esculentus*. *P. lessonae* and *P. esculentus* form stable complexes (L-E complexes) in which *P. esculentus* are hemiclonal. In L-E complexes all the transmitted genomes by *P. esculentus* carry deleterious mutations which are lethal in homozygosity.

**Results:**

We analyze, by means of an individual based computational model, L-E complexes. The results of simulations based on the model show that, by eliminating deleterious mutations, L-E complexes collapse. In addition, simulations show that particular female preferences can contribute to the diffusion of deleterious mutations among all *P. esculentus* frogs. Finally, simulations show how L-E complexes react to the introduction of translocated *P. ridibundus*.

**Conclusions:**

The conclusions are the following: (i) deleterious mutations (combined with sexual preferences) strongly contribute to the stability of L-E complexes; (ii) female sexual choice can contribute to the diffusion of deleterious mutations; and (iii) the introduction of *P. ridibundus* can destabilize L-E complexes.

## Background

Lake frog (*Pelophylax ridibundus* Pallas, 1771) and pool frog (*Pelophylax lessonae* Camerano, 1882) can mate producing the hybrid edible frog (*Pelophylax esculentus* Linneus, 1758). *P. esculentus* can coexist with one or both of the parental species giving rise to mixed populations. Usually, the genotypes of *P. ridibundus*, *P. lessonae* and *P. esculentus* are indicated by *RR*, *LL*, and *LR*, respectively. In Europe there are mixed populations containing *P. ridibundus* and *P. esculentus* individuals, called R-E systems, populations with *P. lessonae* and *P. esculentus* individuals, called L-E systems, and populations with all three species. Due to the eastern origin of *P. ridibundus*, R-E complexes are frequently found in Eastern Europe, while L-E systems are widespread throughout the rest of Europe [1-5]. Hybrids in these populations reproduce in a particular way, called *hybridogenesis*[1,6-12]. Hybridogenesis consists of a gametogenetic process in which the hybrids exclude one of their parental genomes premeiotically, and transmit the other genome, clonally, to eggs and sperm. For example, in L-E complexes, *P. esculentus* hybrids have both the genomes of the parental species, *L* and *R*, but they produce only *R* gametes.

This mode of reproduction requires hybrids to live sympatrically with the parental species whose genome has been eliminated. In this way hybrids in a L-E system eliminate the *L* genome thus producing *P. esculentus* when mating with *P. lessonae*, and generating *P. ridibundus* when mating with other hybrids. Usually *P. ridibundus* generated in L-E complexes are inviable due to deleterious mutations accumulating in the clonally transmitted *R* genome [13-17]. Analogously, in R-E systems there is a tendency during hybrid gametogenesis to eliminate the *R* genome; as with L-E systems, *P. lessonae*, the offspring of hybrids, are often inviable.

In natural L-E complexes, the inviability of offspring of *P. esculentus* ×*P. esculentus* matings is evidenced by the absence of adults *P. ridibundus*. Experimental crosses between coexisting hybrids (from localities sampled throughout the range of L-E populations) also show that such offspring are inviable [6,14,16,17]. These studies have also revealed that the same hybrid individuals, producing inviable progeny, produce viable progeny when crossed with either parental species or with hybrids from different regions. The lethality of natural hybrid × hybrid matings is thus neither the result of hybrid sterility nor the inherent consequence of the hemiclonal reproductive mode. Guex *et al.* present two simple hypotheses, both explaining the observed inviability of *P. esculentus* ×*P. esculentus* progeny by the load of deleterious mutations on the clonally transmitted R genomes [16]. Quoting from their paper, the hypotheses are “(1) inviability is caused by homozygosity for recessive deleterious mutations at particular gene loci; or (2) inviability is caused by a general deterioration of non-recombining *R* genomes through Muller’s ratchet, reflecting different hemiclone-specific sets of incompletely recessive mutations, which leads to lethality when two such deteriorated *R* genomes are combined”. Their conclusion is that the hypotheses are not mutually exclusive, however there is evidence to support the plausibility of the first hypothesis: Muller’s ratchet generates deleterious mutations in relatively random places in the genome, which are then likely to be different in different geographical areas. The study in [16] suggests that, in some cases, single lethal mutations may have the same effect as the accumulation of deleterious ones. However, most studies on *P. esculentus* fitness, suggest that when in a heterozygous state the effect of deleterious mutation is not significant.

Due to the inviability of *P. esculentus* ×*P. esculentus* offspring, *P. esculentus* populations cannot survive alone, but must act as a sexual parasite of one of the parental species. This dependency can be avoided only by all-hybrid populations in which the presence of triploid and tetraploid individuals leads to recombination among homolog parental chromosomes [18-23]. This recombination is able to purge, at least partially, deleterious mutations from genomes. thus producing viable offspring of hybrids. Due to its wide distribution, we will consider L-E complexes, in which *P. esculentus* and *P. lessonae* coexist. In such a complex, the reproductive pattern is shown in Table 1, where the subscript *y* indicates the male sexual chromosome.

**Table 1:** Reproductive pattern of water frogs

	*LL*	*LR*
*L*_*y*_*L*	*L*_*y*_*LLL*	*L*_*y*_*RLR*
*L*_*y*_*R*	*LR*	*RR* not viable

The Y chromosome determines the sex of frog males and can occur only in the L genome, due to primary hybridization which involves, due to size constraints, *P. lessonae* males and *P. ridibundus* females. Only one of the three possible matings resulting in viable offspring produces *LL* genotypes (Table 1). This would give an advantage to *P. esculentus* which could outnumber *P. lessonae* and eventually eliminate them. This situation would also eventually result in an extinction of *P. esculentus* which cannot survive without the parental species. In addition to their relative abundance which is promoted by the above reproductive pattern, *P. esculentus* have other advantages. Although in many cases they show either no differences or intermediate characteristics compared to their parental species [24-27], *P. esculentus* show behavioural differences [28,29], and, have, by heterosis, a greater fitness than the parental species in certain aspects [13,15,30-36]. The combination of the relative abundance and heterosis should out-compete *P. lessonae* in L-E complexes. The widespread distribution of L-E complexes, although with different percentages of hybrids, reveals that there are mechanisms which contribute to the stability of such complexes [37-40]. Of these mechanisms, sexual selection seems to be one of the most important. In fact, *P. esculentus* females prefer (either overtly or cryptically) *P. lessonae* males than males of their own species [41-45]. Many mathematical and computational models have studied the influence of sexual selection in the evolution of populations [46-54]. In addition some models have focused on sexual selection in complexes in which some form of clonal reproduction exists [55-57]. The models in [57-59] show how female preference is able to stabilize L-E complexes by counterbalancing both heterosis and the reproductive advantage of *P. esculentus*. Other factors, such as reproductive performance, in conjunction with sexual choice can increase the stability of L-E complexes [60].

Using an individual-based computational model, in this paper we study three problems. The first is how deleterious mutations contribute to the stability of L-E complexes. The second concerns how, in L-E complexes, deleterious mutations can diffuse in the *R* genomes of the whole *P. esculentus* population. The third is the invasiveness of *P. ridibundus* in L-E complexes.

Regarding the first problem, the aim is to investigate whether deleterious mutations on the *R* genome contribute, together with female preferences, to the stability of L-E complexes.

As for the diffusion of deleterious mutations in the population, an interesting hypothesis is proposed in [61], in which deleterious mutations can influence female preferences. From the literature we know that females of *P. esculentus* have a strong preference for *P. lessonae* males. Vorburger et al. in [61] suggest that, of *P. esculentus* males, *P. esculentus* females may prefer those with mutations on the R genome. Such mutations could make the affected loci on the R genome dysfunctional, thus producing a more “*lessonae*-like” genotype.

Finally, regarding the third problem, Vorburger and Reyer in [9] suggest that the introduction of *P. ridibundus* can either provoke the collapse of L-E populations, or result in a replacement by *P. ridibundus* of both *P. lessonae* and *P. esculentus*, leading to a mono-specific population.

In order to gain further insight into the three problems, we attempt to answer three questions. 

- Is the role of deleterious mutations necessary for the stability of L-E complexes?

- How can a stable L-E complex be obtained?

- What is the effect of introducing *P. ridibundus* into L-E complexes?

## Methods

### The model

To study the interaction between populations of *P. lessonae*, *P. esculentus* and *P. ridibundus* we developed an individual-based model. To answer the three questions above, we started with a simple model (for the first question) and then extended it step by step (to tackle the second and third questions).

In the simplest model, we consider diploid individuals whose genotype is represented by two chromosome types: *L* and *R*. Chromosomes *R* can contain deleterious mutations (represented by *R*_*d*_), and only chromosomes *L* can have the sex-determining chromosome *Y* (represented by *L*_*y*_). Thus the possible genotypes are: *LL*, *L*_*y*_*L*, *LR*, *L*_*y*_*R*, *LR*_*d*_, *L*_*y*_*R*_*d*_, *RR*, *RR*_*d*_, *R*_*d*_*R*_*d*_.

The fitness of a genotype *g*, F(g), is computed as follows:

$$F\left(\;,g\right)=\left\{\begin{array}{ll} 0 & \text{if}\;g=R_{d}R_{d}\\ e^{-\left(\frac{{\left(1-c(\;g)\right)}^{2}}{2\sigma^{2}}\right)} & \text{otherwise} \end{array}\right)$$

where *σ* is a parameter measuring the strength of the ecological selection (smaller values of *σ* correspond to a stronger selection). In the simulations we use two different values for *σ*, *σ*=0.4, which corresponds to a hard environment, and *σ*=0.6, which corresponds to a weaker selection.

Function *c*(*g*), is the *chromosomes fitness*, defined as follows:

$$c\left(\;,g\right)=\left\{\begin{array}{ll} 1 & \text{if}\;g\in \{\text{LR},L_{y}R,{\text{LR}}_{d},L_{y}R_{d}\}\\ 1-\delta_{h} & \text{if}\;g\in \{\text{LL},L_{y}L\}\\ 1-\delta_{h}-\delta_{e} & \text{if}\;g\in \{\text{RR},{\text{RR}}_{d}\} \end{array}\right)$$

where *δ*_*h*_ and *δ*_*e*_ describe the fitness decrement associated with homozygous genotypes (which do not gain from heterosis), and *P. ridibundus* genotypes, respectively. The use of a further decrement, *δ*_*e*_, in the chromosome fitness of *P. ridibundus*, derives from the fact that L-E complexes usually live in pools and marshes, where *P. ridibundus* are less fit. In the following sections, we use *δ*_*h*_=0.2 and *δ*_*e*_ will assume the values 0.0, 0.2, and 0.4. *δ*_*e*_=0.0 means that *P. ridibundus* have the same fitness as *P. lessonae* (i.e. the environment includes niches for both species), while *δ*_*e*_=0.4 represents the fact that *P. ridibundus* are strongly disadvantaged compared to *P. lessonae* (i.e. the environment consists of a typical *P. lessonae* habitat).

We consider that the populations have a reproductive season each year. During this season all the females mate. Female sexual choice is implemented by a *best-of-n* selection procedure of males [52,62], i.e. a female mates with the most preferred of *n* randomly chosen males in the population. The best-of- *n* procedure, which is a usual computational method to take into account female preferences, can be described as follows. Consider a population of males of *k* different types, *m*_1_,*m*_2_,…*m*_*k*_, with numbers of individuals $N_{m_{1}},N_{m_{2}},\ldots N_{m_{k}}$, respectively, and a total male population $N_{m}=N_{m_{1}}+N_{m_{2}}+\ldots +N_{m_{k}}$. Consider a female of type *f* with different preference values for *k* kinds of males, $p{\;}_{m_{1}}^{f},p{\;}_{m_{2}}^{f},\ldots ,p{\;}_{m_{k}}^{f}$, such that $p{\;}_{m_{1}}^{f}<p{\;}_{m_{2}}^{f}<\ldots <p{\;}_{m_{k}}^{f}$. According to the best-of- *n* procedure, the female chooses the best (following her preferences) of the *n* ones randomly taken from the population of all males. Thus the probability, $\text{prob}{\;}_{m_{i}}^{f}$, of a female of type *f*, choosing a male of type *m*_*i*_, *i*∈ [ 0,*k*], is given by the probability of randomly taking no *m*_*j*_ males, with $p{\;}_{m_{j}}^{f}>p{\;}_{m_{i}}^{f}$, times the probability of having at least one *m*_*i*_ male of the taken ones. Using the hypergeometric probability distribution, $\text{pro}b_{m_{i}}^{f}$ is given by the following formula.

probmif=Nm1+Nm2+...+NminNmn∑i=1nNmiiNm1+Nm2+...+Nmi-1n-iNm1+Nm2+...+Nmin

Note that the greater *n* is, the greater the strength of the female preference. Increasing the *n* value will lead to a female choosing from a greater number of males, thus mimicking the behaviour of a more discriminating female. In order to obtain stable complexes, we assume a species-specific female preference, in particular, following the studies in [59,60], we assign a stronger preference to females of the parental species than the hybrid females. *P. esculentus* females have the same behaviour as *P. lessonae*, thus competing for the same kind of males. Likewise, *P. ridibundus* females prefer more “*ridibundus*-like” males. Hereafter we call “*lessonae* preference” this kind of female preference, because *P. lessonae* males are the most preferred. The number of *n* candidates, which each female chooses from, is set to 30, 15, and 30 for the *P. lessonae*, *P. esculentus*, and *P. ridibundus* females, respectively. Choosing from among 30 candidates has a biological flavour. If males are distributed in the environment with a density of one male per square meter, each female must swim in a circle of 10 meters of diameter in order to check out 30 males, a distance which is reasonable for a frog. In [57-59] it is shown that stable populations are only found when the preference of *P. lessonae* females is greater than the preference of *P. esculentus* females. In these papers it is shown that, under the above assumption, many different values of female preference lead to stable complexes. We have performed many simulations by leaving the value of *n* for *P. lessonae* and *P. ridibundus* unmodified, and by varying the value of *n* for *P. esculentus*. We have found that if the ratio of the value of *P. esculentus* to the value of the parental species belongs to the interval [ 0.03,0.7] we obtain stable complexes, when *P. ridibundus* are inviable. This result is analogous to the ones in [57-59]. Because different values in the interval [ 0.03,0.7] affect only the percentage of hybrid frogs in the final stable population, the choice of *n* does not change the overall dynamics of the population. For this reason we use the non-extreme value 0.5.

Offsprings genotypes are obtained from the gamete combination of the parents.

The reproductive season is followed by a viability selection. During this phase the probability that an individual of genotype *g* survives, *p*_*s* *u* *r* *v*_(*g*), is given by a slight modification of the Beverton-Holt model: [63-68]:

$$p_{\text{surv}}\left(\;,g\right)=\frac{1}{1+b\;\phi \;\frac{N}{K(\;g)}}$$

where *b* is the average number of offspring for females that can reach the stage of adults, *ϕ* is the percentage of females in the population, *N* is the number of individuals in the population competing for the resources, and K(g) is the carrying capacity associated with the genotype *g*. K(g) is given by $F(\;g)K_{0}$ where $K_{0}$ is the maximum carrying capacity of the environment. In all our simulations we assume *b*=6 and $K_{0}=3000$. The standard Beverton-Holt model is modified due to the fact that we consider overlapping generations and we apply the viability selection based on survival probability, not only to young tadpoles but to all the individuals in the population. The simplest model is used to answer the first question by performing both simulations in which all *R* genomes carry deleterious mutations and simulations in which all *R* genomes are free from deleterious mutations.

To answer the second question, “How can a stable L-E complex be obtained?”, we simulate the diffusion of deleterious mutations in the population, starting with an L-E complex, composed by *P. lessonae*, in which there are only a few *P. esculentus* individuals (without mutations on the *R* genome). We consider a mutation rate, *μ*, which gives the probability of adding a new deleterious mutation on the *R* genome of an offspring (in the simulations *μ* is set both to 10^-4^ and to 10^-5^). We consider different “stages” in the accumulation of mutations in the *R* genome. For the sake of simplicity we consider only three stages: *R*, *R*_*d*1_, and *R*_*d*_. *R* is the genome without mutations, *R*_*d*1_ is the genome with a non lethal accumulation of mutations, and, as before, *R*_*d*_ is the final stage of accumulation (lethal in homozygous individuals). With this scenario we have *P. ridibundus* females with the following possible genotypes (in order of decreasing fitness): *RR*, *RR*_*d*1_, *RR*_*d*_, *R*_*d*1_*R*_*d*1_, *R*_*d*1_*R*_*d*_, *R*_*d*_*R*_*d*_, where *R*_*d*_*R*_*d*_ is the only lethal genotype. The fitness decrease of each genotype with respect to the previous one is given by *δ*_*m*_. In the simulations we set *δ*_*m*_=0.04. In this scenario, following [61], we assume that *P. lessonae* and *P. ridibundus* females have a strong preference for males of their own species, while of *P. esculentus* males, females prefer males with a more “*lessonae*-like” genotype (males with mutations on the *R* genotype). We call this kind of female preference “*lessonae*-like preference”. We have *P. esculentus* males with the following genotype: *L*_*y*_*R*, *L*_*y*_*R*_*d*1_, *L*_*y*_*R*_*d*_, which, according to the “*lessonae*-like preference”, are in order of increasing preference by the female.

Finally, to answer the third question, “What is the effect of introducing *P. ridibundus* into L-E complexes?”, we need to simulate the effect of the introduction of *P. ridibundus* males and females in an L-E complex. In these simulations we consider both *R* genomes without mutations; this is because we assume the absence of the Muller ratchet in the (sexually reproducing) introduced *P. ridibundus* and *R*_*d*_ genomes generated by the stable L-E complex. The further extension we consider is the possibility of having *P. ridibundus* males, i.e. the possibility of having the *Y* chromosome on *R* genomes as well: *R*_*y*_ and *R*_*y* *d*_.

We introduced a limit for the lifespan of individuals, thus all individuals exceeding 10 years of age are removed from the population. Removing old frogs from the system avoids that extremely fit individuals survive indefinitely because the viability selection is not able to remove them. This situation can easily happen in models with overlapping generations which do not consider deaths due to aging.

The parameters used in the model, with their meaning and values, are reported in Table 2.

**Table 2:** Parameters used in the model

*σ*	Selection strength	0.4-0.6
*δ*_*h*_	Fitness decrease due to homozygosity	0.2
*δ*_*e*_	Fitness decrease for environmental reasons	0.0-0.4
*δ*_*m*_	Fitness decrease for each step of mutation accumulation	0.04
*n*	Number of attempts per female in choosing males, *P. lessonae*, *P. esculentus*, *P. ridibundus*	30, 15, 30
*b*	Number of surviving tadpoles for each female	6
$K_{0}$	Environmental carrying capacity	3000
*μ*	mutation rate	10^-5^, 10^-4^

#### Implementation

The process was formalize with CLS [69-75] and then implemented in C++ language using Microsoft Visual Studio 2010. Each simulation runs for 2000 iterations (more when necessary) with a carrying capacity of 3000 individuals. A single simulation takes nearly 10sec on a worstation equipped with Intel i5 3.2Ghz processor and 8GB RAM.

## Results

### Is the role of deleterious mutations crucial for the stability of L-E complexes?

**Figure 1:**
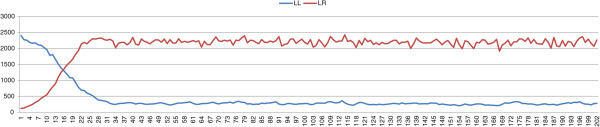
**Results with an initial population composed of *P. lessonae * frogs and a percentage of 5% *P. esculentus *carrying deleterious mutations. ***σ*=0.4, *μ*=0.

The first step in studying the role of deleterious mutations for the stability of L-E complexes is to consider an initial population composed of *P. lessonae* frogs and small percentages of *P. esculentus*: 5*%*, 10*%*, and 20*%*, respectively. Note that an initial situation with a large number of *P. lessonae* individuals favours the stabilization of the complex (there is no possibility of an early collapse due to the greater fitness of *P. esculentus*). We assume that all *P. esculentus* individuals carry the deleterious mutations on the *R* genome, that is *P. ridibundus* females are not viable and they do not appear in the population. We assume the “*lessonae* preference” for females, based on the values 30, 15, and 30 for the best-of- *n* procedure for the three species, as described in the previous section. Finally we consider *δ*_*h*_=0.2. We perform simulations with *σ*=0.4 and *σ*=0.6. For each combination of parameters *σ* and *δ*_*e*_ we performed 100 simulations, the possible outcomes of which are either a stable L-E complex or the collapse of the whole population. In all the simulations the populations evolve towards a stable L-E complex, following a typical population composition pattern (Figure 1).

The result of these simulations is not surprising. Essentially the results are in accordance with those in [58,59], showing that female preference is a strong stabilizing force for L-E complexes.

**Figure 2:**
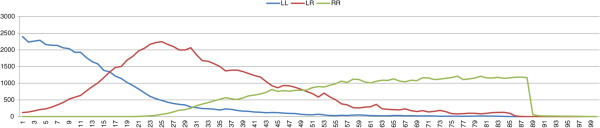
**Results of typical simulations with an initial population composed of *P. lessonae *frogs and a percentage of 5% mutation-free *P. esculentus*.***σ*=0.4, *δ*_*e*_=0.2, *μ*=0.

In order to investigate the role of deleterious mutations in the stability of L-E complexes, we consider the same parameters as the previous simulations, but we remove the deleterious mutations from all the *R* genomes of the initial population. In addition, we set *δ*_*e*_ to 0.2 and 0.4, that is we consider that all *P. ridibundus* born from hybrids are always disadvantaged compared to *P. lessonae* and *P. esculentus*. We also set the mutation rate *μ* equal to 0, in order to prevent deleterious mutations, considering only a “mutation-free” population. We can observe that with all the parameter combinations and the initial percentages of *P. esculentus*, in all the simulations the population eventually collapses. If viable *P. ridibundus* females are produced, the reproductive pattern becomes as depicted in Table 3. The table highlights that this reproductive pattern generates a numerical disadvantage for *P. lessonae*, the population of which decreases. The decrease in the *P. lessonae* population has, as a consequence, a decrease in produced *L* gametes, which, in turn, results in a higher production of *P. ridibundus*. Thus the population of *P. ridibundus* females grows and eventually out-competes the other species, despite the weaker fitness of *P. ridibundus* females compared both to *P. esculentus* (*δ*_*h*_=0.2) and to *P. lessonae* (*δ*_*e*_=0.2 and *δ*_*e*_=0.4), see Figure 2. Of course a population of only *P. ridibundus* females cannot survive.

**Table 3:** Reproductive pattern of water frogs without deleterious mutations

	*LL*	*LR*	*RR*
*L*_*y*_*L*	*L*_*y*_*LLL*	*L*_*y*_*RLR*	*L*_*y*_*RLR*
*L*_*y*_*R*	*LR*	*RR*	*RR*

Note that the higher the percentage of *P. esculentus* in the initial population, the smaller the number of generations before the collapse. By varying either the strength of female preferences or the strength of ecological selection, we obtain different values for the number of generations before the collapse, but an evolution towards collapse remains the overall trend of the system. Even when *P. ridibundus* are at a serious disadvantage, *δ*_*e*_=0.4, *RR* females are able to compromise the stability of L-E complexes. Of course, we amplify this effect by decreasing *δ*_*e*_, thus we do not show the population dynamics with *δ*_*e*_=0.0. In this case, the point of collapse is reached very quickly.

### How can a stable L-E complex be obtained?

In this section we study the effect of both “*lessonae* preference” and “*lessonae*-like preference” of *P. esculentus* and *P. lessonae* females in the diffusion of deleterious mutations. We set the mutation rate, *μ*, either to 10^-4^ or to 10^-5^. We consider three possible stages of deleterious mutation accumulation in each *R* genome (*R*, *R*_*d*1_, and *R*_*d*_), thus any mutation event determines the passage from one stage to the next. We start the simulations with two different initial populations. The consistency of the initial *P. lessonae* population is the same in all the simulations, 2700 individuals, but the number of *P. esculentus* individuals is set, initially, either to 10 or 100 individuals. We also perform simulations with two values for *δ*_*e*_, which lead to a decrease in fitness of mutation-free *P. ridibundus* compared to *P. lessonae*. We set *δ*_*e*_ to either 0.2 or 0.4, i.e. we consider that the environment is either weakly penalizing for *P. ridibundus* or is a typical *P. lessonae* habitat, which is not suitable for *P. ridibundus* frogs. In addition, any further mutation accumulation on the *R* genomes of *P. ridibundus* decreases their fitness by *δ*_*m*_=0.4.

**Figure 3:**
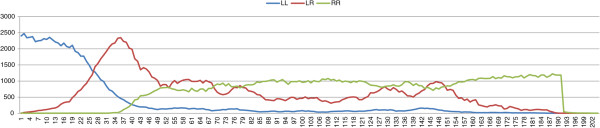
**Results of typical simulations with an initial population composed of *P. lessonae * frogs and 10 mutation-free *P. esculentus*.** In the simulations we assume a “*lessonae* preference” for *P. esculentus* and *P. lessonae* females. *σ*=0.4, *δ*_*e*_=0.4, *μ*=10^-4^.

**Figure 4:**
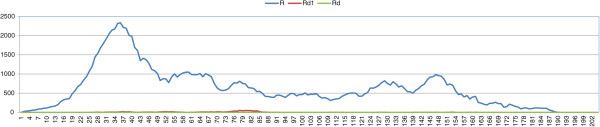
**Results of typical simulations with an initial population composed of *P. lessonae * frogs and 10 mutation-free *P. esculentus*.** In the simulation we assume a “*lessonae* preference” for *P. esculentus* and *P. lessonae* females. *σ*=0.4, *δ*_*e*_=0.4, *μ*=10^-4^. *R*, *R*_*h*_*d*, and *R*_*d*_ are the number of *R* genomes in the *P. esculentus* population with three different levels of mutation accumulations.

Firstly, we consider the “*lessonae* preference”. According to this preference pattern, *P. esculentus* and *P. lessonae* females prefer *P. lessonae* males, and, do not choose among *P. esculentus* males. The results of the simulations show that in our model both with 10 and with 100 *P. esculentus* mutation-free individuals in the initial population, and with any value for *δ*_*e*_ and *σ*, the lethal deleterious mutation diffuses slowly. This slow diffusion is not able to prevent the production of a sufficient number of viable *P. ridibundus* females, which leads to the collapse of the population with all possible parameter values. Figure 3 shows a typical dynamic of the population while Figure 4 shows the diffusion of mutations in the *R* genome of *P. esculentus*. The results of the simulations with *σ*=0.4 and *μ*=10^-4^ are shown in Table 4.

**Table 4:** **Outcomes of 100 simulations with an initial *P. esculentus * population of 10 and 100 individuals without deleterious mutations on the *R *** **genome**

**a)** ***δ***_***e***_**=0** ***.*** **2**		
Initial *P. esculentus* pop.	10	100
Stable L-E system	0	0
LL population	0	0
Collapse	100	100
**b)** ***δ***_***e***_**=0** ***.*** **4**		
Initial *P. esculentus* pop.	10	100
Stable L-E system	0	0
LL population	0	0
Collapse	100	100

In the simulations we assume a “*lessonae* preference” for *P. esculentus* and *P. lessonae* females. The parameters are: *σ*= 0.4, *μ*= 10^-4^.

**Figure 5:**
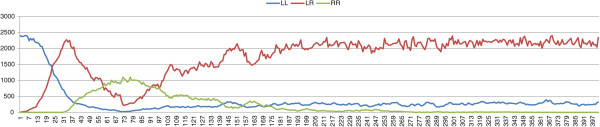
**Results of typical simulations with an initial population composed of *P. lessonae * frogs and 10 mutation-free *P. esculentus*.** In the simulations we assume a “*lessonae*-like preference” for *P. esculentus* and *P. lessonae* females. *σ*=0.4, *δ*_*e*_=0.4, *μ*=10^-4^.

**Figure 6:**
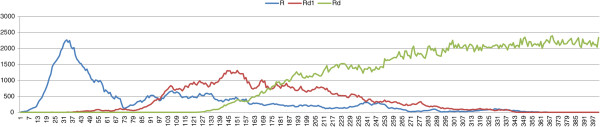
**Results of typical simulations with an initial population composed of *P. lessonae * frogs and 10 mutation-free *P. esculentus*.** In the simulation we assume a “*lessonae*-like preference” for *P. esculentus* and *P. lessonae* females. *σ*=0.4, *δ*_*e*_=0.4, *μ*=10^-4^. *R*, *R*_*h*_*d*, and *R*_*d*_ are the number of *R* genomes in the *P. esculentus* population with three different levels of mutation accumulations.

Secondly, we consider the “*lessonae*-like preference”. According to this preference pattern *P. esculentus* and *P. lessonae* females prefer *P. lessonae* males, and, of *P. esculentus* males, they prefer those with a greater accumulation of deleterious mutations. The results of simulations confirm that female choice can lead to the diffusion of deleterious mutations in the *P. esculentus* genomes if those mutations silence *P. ridibundus* traits and result in more “*lessonae*-like” *P. esculentus* males. In this scenario, stable complexes can be obtained with *δ*_*e*_=0.4, while a higher fitness of *P. ridibundus* leads to the extinction of the population (Table 5). Starting with mutation-free *P. esculentus* individuals, the diffusion of mutations passes through different stages of mutation accumulations. The pattern of mutation diffusion is that mutation rates contribute to deleterious mutations, while the “*lessonae*-like preference” favours the production of offspring of *P. esculentus* males with a stronger mutation accumulation on their *R* genome. In the progression of mutation accumulation, *RR* genotypes gets more and more inviable while *LR* males become more and more attractive. When *δ*_*e*_=0.4, Figure 5 shows a typical dynamic of the population, while Figure 6 shows the diffusion of mutations in the *R* genome of *P. esculentus*. The results of the simulations with *σ*=0.4 and *μ*=10^-4^ are shown in Table 5.

**Table 5:** **Outcomes of 100 simulations with an initial *P. esculentus * population of 10 and 100 individuals without deleterious mutations on the *R *** **genome**

**a)** ***δ***_***e***_**=0** ***.*** **2**		
Initial *P. esculentus* pop.	10	100
Stable L-E system	0	0
LL population	0	0
Collapse	100	100
**b)** ***δ***_***e***_**=0** ***.*** **4**		
Initial *P. esculentus* pop.	10	100
Stable L-E system	76	73
LL population	1	0
Collapse	23	27

In the simulations we assume a “*lessonae*-like preference” for *P. esculentus* and *P. lessonae* females. The parameters are: *σ*= 0.4, *μ*= 10^-4^.

**Table 6:** **Outcomes of 100 simulations in which in a stable L-E complex a percentage of 5% of *P. ridibundus*, males and females, are introduced**

	***δ***_***e***_**=0** ***.*** **0**	***δ***_***e***_**=0** ***.*** **2**	***δ***_***e***_**=0** ***.*** **4**
Stable L-E system	0	46	100
Stable L-E-R system	32	0	0
Stable *P. ridibundus* population	39	0	0
Collapse	29	54	0

The three columns correspond to different values of *δ*_*e*_, 0.0, 0.2, and 0.4, respectively. *σ*= 0.4, *μ*= 0.

### What is the effect of the introduction of *P. ridibundus* in L-E complexes?

In this section we analyze the effect of introducing *P. ridibundus* into an L-E complex. This scenario really happens in natural environments due to the importation in Western Europe of *P. ridibundus* for commercial purposes.

In order to study the effect of the translocation of *P. ridibundus*, we performed simulations by varying the fitness of the introduced frogs (*δ*_*e*_=0.0, 0.2, 0.4). *δ*_*e*_=0.0 means that the environment does not put *P. ridibundus* at a disadvantage with respect to *P. lessonae*. In these simulations we consider a strong selection strength, *σ*=0.4.

**Figure 7:**
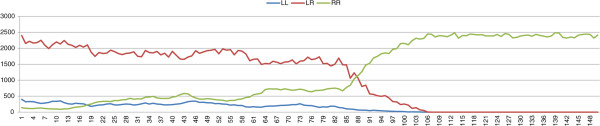
**Results of typical simulations with an initial stable L-E population in which a percentage of 5% of *P. ridibundus*, males and females, are introduced.***σ*=0.4, *δ*_*e*_=0.0, *μ*=0.

**Figure 8:**
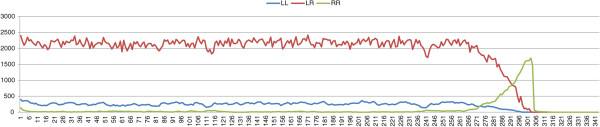
**Results of typical simulations with an initial stable L-E population in which a percentage of 5% of *P. ridibundus*, males and females, are introduced.***σ*=0.4, *δ*_*e*_=0.2, *μ*=0.

**Figure 9:**
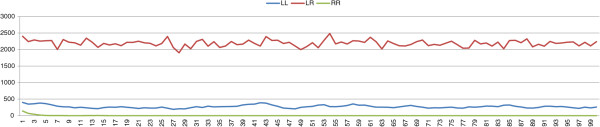
**Results of typical simulations with an initial stable L-E population in which a percentage of 5% of *P. ridibundus*, males and females, are introduced.***σ*=0.4, *δ*_*e*_=0.4, *μ*=0.

We study the effect of introducing, in a stable L-E complex, a percentage of both 5*%* and 10*%* of *P. ridibundus*, males and females. We consider three different situations: i) *P. ridibundus* are introduced into the typical environment for *P. lessonae* (*δ*_*e*_=0.4), ii) *P. ridibundus* are introduced into a mixed (lake/marsh) environment (*δ*_*e*_=0.2), and iii) *P. ridibundus* are released into an environment that is fit for them (possibly close to resident frogs) (*δ*_*e*_=0.0). We have four possible outcomes: stable L-E systems, stable L-E-R systems, stable *P. ridibundus* populations, and the collapse of the population (Table 6, Figures 7, 8, and 9). In the figures we show the dynamics for *σ*=0.4. For *σ*=0.6 we obtain analogous evolutions.

If the fitness of *P. ridibundus* is equal to the fitness of *P. lessonae* (*δ*_*e*_=0.0), in most cases the population becomes a mono-specific population of *P. ridibundus*. Note that there are many cases in which the three species coexist at the end of the simulations, which we discuss explicitly in the following section. If the fitness of *P. ridibundus* decreases (*δ*_*e*_=0.2), the introduced frogs do not survive for long. However, before their extinction *P. ridibundus* can mate with *P. lessonae* and *P. esculentus*, thus introducing mutation-free *R* genomes into the hybrid population. At this point, viable *P. ridibundus* females born from matings between hybrids lead the population to collapse. Finally, when the fitness of *P. ridibundus* is very low (*δ*_*e*_=0.4), *P. ridibundus* frogs are immediately expelled from the system.

## Discussion

From the results of the previous section we can deduce the overall dynamics of L-E complexes with viable *P. ridibundus* females. In general, the presence of viable *P. ridibundus* females significantly changes the reproductive outcome of L-E complexes, and the generation of offspring becomes as depicted in Table 3. For the sake of simplicity let us consider hypothetical complexes in which the number of females is the same for the three species. For such populations the production of *P. lessonae* offspring passes from 33.33*%* in stable L-E complexes (recall that in such complexes *P. ridibundus* are inviable and do not survive) to 16.66*%* in complexes where *P. ridibundus* survive. Thus the viability of *P. ridibundus* decreases the relative abundance of *P. lessonae* offspring. The decrease in the relative production of *P. lessonae* offspring leads to a decrease in *P. lessonae* adults in the future. This, in turn, causes a decrease in the production of *L* gametes, which are produced only by *P. lessonae* frogs. This process, if not stopped by some external reasons, results in a trend towards the extinction of *P. lessonae*. A population composed only of *P. esculentus* individuals and *P. ridibundus* females cannot survive, because no gametes with the *Y* chromosome can be generated.

The discussion which follows, regarding the three questions mentioned previously, is based on the trend towards extinction mentioned above, modulated for example by the accumulation of mutations, or the introduction of viable *P. ridibundus* males.

### Deleterious mutations are necessary for the stability of L-E complexes

We showed in Section ‘Is the role of deleterious mutations crucial for the stability of L-E complexes?’ that by using the “*lessonae* preference”, we essentially obtain the same results as [58,59]. If there are no viable *P. ridibundus* offspring, the system evolves towards stability regardless of the strength of the selection. The same complexes, with the same values of female preferences, but without deleterious mutations on *R* genomes will collapse irrespectively both of the selection strength and the fitness of viable *P. ridibundus*. In the dynamics of the population towards collapse, there are roughly three phases. In the first phase, the effect of sexual selection and the abundance of *P. lessonae* males maintains a low number of *P. ridibundus* offspring. Although no mutations are present in the *P. esculentus* genome, a very few *P. ridibundus* females are generated because mating between hybrids is rare, due to female preferences. In the second phase, the number of hybrids increases because of their fitness as a result of heterosis, while the number of *P. lessonae* decreases. In this phase the increased number of hybrids facilitates mating which thereby produces viable *P. ridibundus* females. In the third phase, *P. ridibundus* females act as sexual parasites for hybrids, and their number grows because of *P. esculentus* ×*P. esculentus* and *P. esculentus* ×*P. ridibundus* matings. Note that these matings produce only *P. ridibundus* females. Viable *P. ridibundus* females will mate, preferably, with *P. esculentus* males (which have a more “*ridibundus* phenotype”) producing a bigger population of *ridibundus* females for any further generation. In the model we do not consider male frogs as a limiting factor for reproduction, that is one male is able to fertilize the eggs of a unlimited number of females. We consider also that males will not make any specific choice of females [45]. The number of *P. esculentus* decreases because of the reduced number of *P. lessonae*. This phase ends with a population of all *P. ridibundus* females which quickly collapses (Figure 2).

Consequently, if stability is maintained by female preferences, collapse only can be prevented if the initial population of *P. esculentus* is affected by deleterious mutations on the *R* genomes, which prevents the birth of viable *P. ridibundus*. Thus we can conclude that, in a system in which the parameters used for the stability are essentially female preferences and viability of *P. ridibundus*, deleterious mutations are necessary for the stability of the complex. These results help to clarify why natural L-E complexes generating viable *P. ridibundus* are extremely rare, and in most cases the percentage of viable *P. ridibundus* is not significant [15]. It is difficult (perhaps impossible) for L-E complexes to persist if they generate viable *P. ridibundus*. Starting with an initial population in which all *P. esculentus* frogs carry deleterious mutations, the complex evolves towards a stable configuration (Figure 1). The possible collapse of the population occurs only if *P. esculentus* out-compete *P. lessonae*, however this evolution seldom takes place because of the large number of *P. lessonae* in the initial population. The results show that, with the same selection strength, L-E complexes evolve towards the same percentages of the two frog species, whatever the initial percentages.

Our results regarding the stability of L-E complexes both with deleterious mutations in all *R* genomes and with a “*lessonae* preference” are similar to those in [57-59]. We show that deleterious mutations strongly influence the stability of L-E complexes. Thus deleterious mutations are not only a secondary consequence of Muller’s ratchet but they have an important role in the stability of complexes. In this paper we highlight that, if we consider both heterosis and females preferences as the only stabilizing forces of L-E complexes, the lack of deleterious mutations drives such populations to collapse. Thus neither female preferences nor deleterious mutations are sufficient to maintain the stability of L-E complexes however, in this scenario, each is necessary for stability.

### Female preference can contribute to obtaining stable L-E complexes

The above discussion highlights the important role of deleterious mutations in stable L-E complexes. But how can stable L-E complexes be obtained?

Our results show that, in order to reach stable L-E populations, there must be forces that drive the diffusion of deleterious mutations. Even with a fast mutation rate, 10^-4^, Muller’s ratchet alone is not sufficient for diffusing the deleterious mutations in the population. The “*lessonae* preference” does not allow *P. lessonae* and *P. esculentus* females to choose from among *P. esculentus* males. Thus the diffusion of deleterious mutation among *P. esculentus* individuals and *P. ridibundus* females is not guided by female preferences, but only by the mutation rate (essentially by Muller’s ratchet). In all our simulations this diffusion turns out to be very slow, thus viable *P. ridibundus* females are generated before Muller’s ratchet accumulates lethal mutations on all *R* genomes. This intermediate phase, with a sufficient number of viable *P. ridibundus* females, is responsible for the collapse of the whole system.

Selecting *R* genomes with a higher mutation accumulation is accelerated by the “*lessonae*-like preference”. In this case the production of offspring with greater mutation accumulation on the *R* genome is favoured, and consequently the production of fit *P. ridibundus* females is lowered. To understand this process we need to consider that mutation accumulation on *R* genomes decreases the fitness of *P. ridibundus* females, but it does not affect the fitness of *P. esculentus* in which dysfunctional *R* genomes are counterbalanced by “healthy” *L* genomes.

Another important point is that a significant parameter in the diffusion of deleterious mutations is the fitness of *P. ridibundus* to the environment. If this fitness is too high, too many viable *P. ridibundus* females are produced before significant mutation accumulations. We know that a high number of such viable females will lead the population to collapse.

For computational purposes we have considered only three stages of mutation accumulation on the *R* genomes. This is an approximation of the Muller’s ratchet which, in most cases, operates through a huge number of mutations. We approximate the Muller’s ratchet by decreasing the mutation rate so that a mutation in the model corresponds to many mutations in real genomes. Following the estimation in [76], we assume that, in an eukaryote organism, the mutation rate in a whole genome during sexual reproduction is in the interval [3×10^-2^,9×10^-1^]. Many of these mutations are either not significant or not deleterious. Values of the mutation rate in the interval [10^-5^,10^-4^], used in our model, take both the above considerations into account.

Our study differs significantly with the results of other authors with regard to the diffusion of mutations. The models in [58-60] provide an extensive insight into the reasons for the stability of L-E complexes starting from the assumption that deleterious mutations are present in all the *R* genomes in the population. Our model builds on the previous ones by assuming sexual choices in the populations. However, it differs by considering a population in which deleterious mutations are not present, but are generated according to a mutation rate. In addition, only when this accumulation reaches a given threshold does it become lethal. This leads us to conclude that sexual selection not only stabilizes the complexes, but can also force mutation diffusion.

Note that our simulations do not enable us to prove the hypothesis suggested in [61]. Computational and mathematical models, without subsequent experimental support, can only be used to rule out incorrect hypotheses - they cannot prove correct ones. Computational and mathematical models can only state that a hypothesis is plausible. In the case of L-E complexes, the stabilization period is so long that no real experiment can support a hypothesis on its stabilization, however our model suggests that the hypothesis in [61] regarding female preference, could plausibly lead to stable L-E complexes.

### Invasion of translocated *P. ridibundus*

Another point that we study with our model is the consequence of introducing *P. ridibundus* into stable L-E complexes. *P. ridibundus* can mate both with *P. esculentus*, producing *P. ridibundus*, and with *P. lessonae* (primary hybridization), producing *P. esculentus*. Primary hybrids can have low fertility rates [77], thus their contribution to the dynamics of the population is low. In our model we take account of this low contribution by decreasing the possibility of producing primary hybrids. This is done by setting the female preferences of *P. ridibundus* in a way that *P. lessonae* males are seldom chosen (the value of *n*, in the best-of- *n* procedure, is set to 30). On the other hand, the preference of *P. lessonae* females is mainly for males of their own species. We assign the same fitness both to the introduced *P. ridibundus* and to those generated by matings of *P. ridibundus* with *P. esculentus*. This is in accordance with the semi-natural experiments in [17]. However, in some simulations we use a *P. ridibundus* fitness, which is lower than the fitness of the resident *P. lessonae* and *P. esculentus* because we consider that a marshy environment with a low oxygen level, where *P. lessonae* and *P. esculentus* live, is less suitable for *P. ridibundus*.

The results show that, as predicted in [9], the introduced *P. ridibundus* often out-compete the other species resulting in a mono-specific population, when their fitness is comparable to the resident population’s. Although the introduction of *P. ridibundus* results in new *R* hemiclones, which contribute to the genetic diversity of hybrids, our results do not support the hypothesis presented in [5] that this genetic diversity can stabilize the hybridogenetic system. If the fitness of *P. ridibundus* is competitive with the fitness of the resident population (*δ*_*e*_=0.0), *P. ridibundus* males will survive and *P. ridibundus* often will replace the original population. Note that in this case we also have stable L-E-R complexes as the outcome. This is a system where two independent populations coexist, an L-E complex and a *P. ridibundus* population. The L-E complex is stable due to female preferences and lethal mutations on the *R* genomes, while the *P. ridibundus* population is stable because of the absence of deleterious mutations, which are purged by selection. The two populations do not cross because the number of individuals in both is high enough to ensure that females of one population in most cases find a preferred male, of the same population, in the set randomly chosen by the best-of- *n* procedure.

The whole population collapses when the introduced frogs have a low fitness. In this case, *P. ridibundus* individuals will not survive for long, given their unfitness, but *P. ridibundus* frogs, before their death, can introduce *R* genomes without mutation in the *P. esculentus* population, thus provoking the collapse of the complex.

Finally, by assuming that *P. ridibundus* are at a considerable disadvantage, the introduced unfit population is out-competed. During their short survival time, *P. ridibundus* females are not able to have a sufficient number of matings with *P. esculentus* males, thus they cannot introduce a sufficient number of *R* genomes without mutations into the *P. esculentus* population.

## Conclusions

We have presented an individual-based computational model to study L-E water frog populations, i.e. complexes composed of *P. lessonae* and *P. esculentus*. The individual based model considers not only the genotypes, but also the age of each individual and the average lifespan. In addition, female preferences (implemented by a best-of- *n* procedure) and ecological selection are considered.

We believe that our results highlight that: 

- deleterious mutations in the *R* genomes strongly contribute, together with sexual preferences, to maintaining the stability of such complexes,

- female sexual choice can contribute to the diffusion of *R* genomes carrying deleterious mutations in L-E complexes, and

- the introduction of *P. ridibundus* can destroy L-E complexes.

## Competing interests

The authors declare that they have no competing interests.

## Authors’ contributions

All authors conceived the computational model. PB programmed the simulator and performed the experiments. All authors contributed to write the paper. All authors read and approved the manuscript.
